# Methods for the Refinement of Protein Structure 3D Models

**DOI:** 10.3390/ijms20092301

**Published:** 2019-05-09

**Authors:** Recep Adiyaman, Liam James McGuffin

**Affiliations:** School of Biological Sciences, University of Reading, Reading RG6 6AS, UK; r.adiyaman@pgr.reading.ac.uk

**Keywords:** protein model refinement, tertiary structure prediction, molecular dynamics simulations, energy functions, model quality estimates, Critical Assessment of techniques for Structure Prediction (CASP)

## Abstract

The refinement of predicted 3D protein models is crucial in bringing them closer towards experimental accuracy for further computational studies. Refinement approaches can be divided into two main stages: The sampling and scoring stages. Sampling strategies, such as the popular Molecular Dynamics (MD)-based protocols, aim to generate improved 3D models. However, generating 3D models that are closer to the native structure than the initial model remains challenging, as structural deviations from the native basin can be encountered due to force-field inaccuracies. Therefore, different restraint strategies have been applied in order to avoid deviations away from the native structure. For example, the accurate prediction of local errors and/or contacts in the initial models can be used to guide restraints. MD-based protocols, using physics-based force fields and smart restraints, have made significant progress towards a more consistent refinement of 3D models. The scoring stage, including energy functions and Model Quality Assessment Programs (MQAPs) are also used to discriminate near-native conformations from non-native conformations. Nevertheless, there are often very small differences among generated 3D models in refinement pipelines, which makes model discrimination and selection problematic. For this reason, the identification of the most native-like conformations remains a major challenge.

## 1. Introduction

The determination of three-dimensional protein structures at an atomic resolution is the key to unlocking an understanding of biological functions and the molecular mechanisms of diseases [1,2]. Although the established experimental methods, such as X-ray crystallography [3,4,5,6,7], Nuclear Magnetic Resonance (NMR) [8,9], and cryo-electron microscopy [9,10], may enable the determination of 3D atom coordinates at high accuracies, they are far from matching the pace of new genetic data, due to their high cost and laborious processes in the cloning, expression, and purification stages [11,12,13,14]. Accurate *in silico* protein modelling is comparatively cheaper and faster than experimental determination methods, and helps us to bridge the gap between the known sequences and available structures. Furthermore, *in silico* modelling is often able to provide detailed structure representations at an atomic level [1,2,15,16,17,18,19,20].

*In silico* prediction of protein structures consists of three main stages, starting with: (1) predicting 3D models by template-based modelling (TBM) and free modelling (FM); continuing with (2) the assessment of the predicted 3D models; and ending with (3) the refinement of the predicted 3D models [16,21]. The prediction of 3D models from amino acid sequences has made significant progress towards the accurate determination of native structures, especially with the use of templates from known structures of homologous proteins, and the progress has been well-documented in the last 25 years of the CASP experiments [22,23,24,25,26,27]. In general, 3D modelling can be divided into two broad categories (in terms of the usage, or not, of a known template structure): TBM and FM [16]. TBM [26,28,29] methods are able to generate reliable 3D models, based on the available known structures, by copying the relative atom coordinates from the aligned residues through sequence-structure alignments; such approaches have been found to be the most successful for tertiary protein structure prediction, by far [5,18,30,31,32]. If there is a high similarity between the target sequence and the template from the protein data bank [33,34], then the predictions are likely to be highly accurate [18,21,30,35]. In addition, the increasing number of available structures determined by advanced experimental techniques allows for an increasingly higher coverage of protein structures [36,37,38,39].

In the cases where no suitable templates are available for generating predicted 3D models, then template-free modelling (FM), or ab initio modelling, is used to predict the models by relying on physical, chemical, and thermodynamic principles [16]. However, the accuracy of the 3D models produced by FM has often been much lower than those produced by TBM and, historically, FM methods have only been accurate in modelling small protein structures, up to 100 residues [16]. TBM and FM approaches may generate hundreds of 3D structure “decoys” in different alternative conformations [16,40]. Model Quality Assessment Programs (MQAPs) have been used to determine the most native-like 3D model among the decoys, by giving local and global scores, which can be used to estimate model accuracy [12,41,42,43].

The accuracy of the predicted 3D models is a critical factor for detailed mechanistic studies, such as drug design, protein docking, and the prediction of protein function. Furthermore, pharmaceutical applications often require structures close to experimental levels of accuracy [5,30,32,44,45,46,47,48,49,50,51]. Although the success of TBM and FM modelling has been observed in the CASP experiments, often the predicted 3D models are not without flaws—particularly those from FM methods—and they may still have some local and global errors, including: irregular contacts or hydrogen bonds, clashes, and unusual bond angles and lengths in the predicted 3D models [26,42,52,53]. The errors in the predicted 3D structures also limit the usage of the models for further studies. The necessity for increasing the accuracy of the predicted tertiary structures and the correction of the errors described above has led to development of methods for the refinement of 3D models [5,43,54].

The refinement of 3D models of proteins has emerged as the last milestone of the structure prediction journey to reach parity with experimental accuracy [55,56]. Refining 3D models often helps to bring them closer to native structures by modifying the secondary structure units and repacking sidechains [54]. However, ironically, refinement approaches can also lead to a degradation in the quality of models. Knowing whether a model has been improved or made worse remains a major challenge for developers of 3D model refinement methods [57,58]. Consistent beneficial refinement of predicted 3D models is necessary for many *in silico* studies, ranging from drug discovery to protein design [47,50,59,60,61,62,63]. 

**Figure 1:**
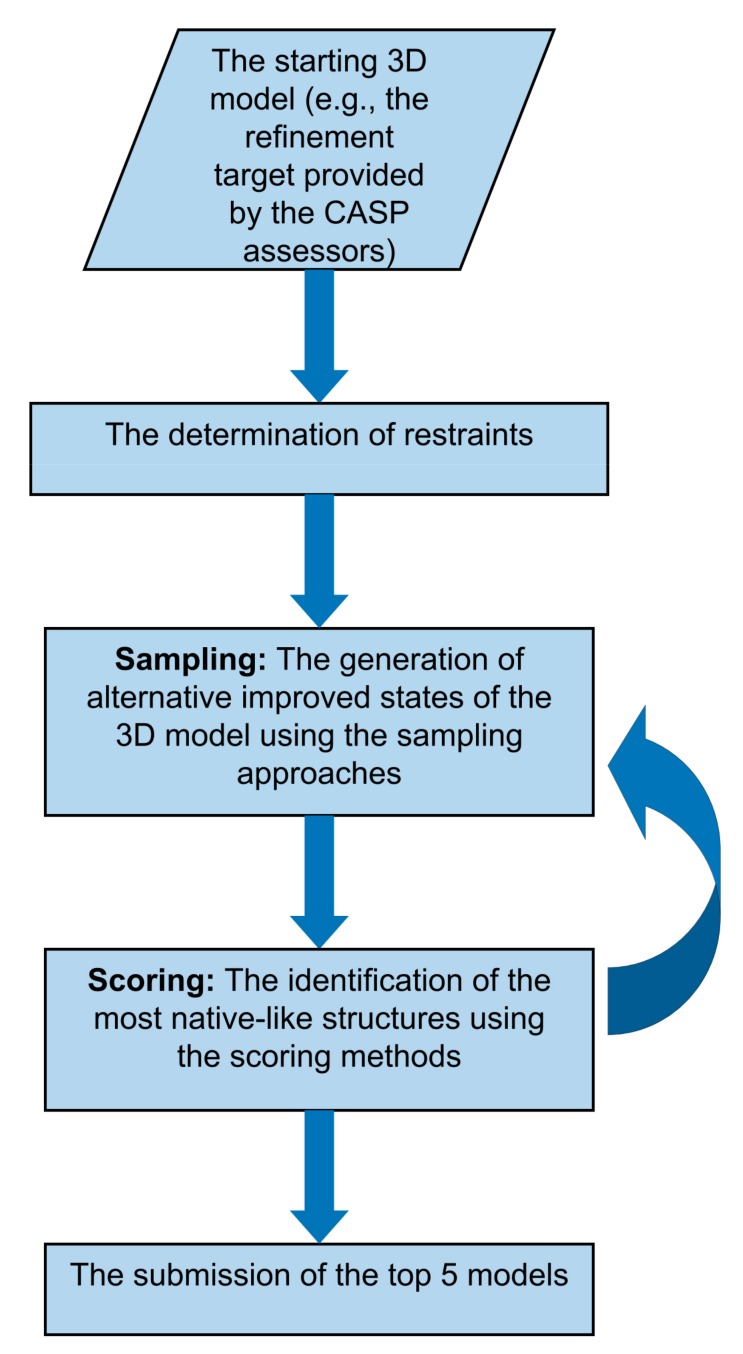
Flowchart outlining the generalized protocol for the refinement of tertiary structure models applied by groups during the Critical Assessment of techniques for Structure Prediction (CASP) experiments.

Typically, the refinement of predicted 3D models involves two principal stages: Sampling and scoring [5,53] (see Figure 1). For successful refinement, firstly, the sampling approaches have to be able to generate at least some alternative 3D models that are closer to the native structure than the initial model and, secondly, the generated 3D models must be accurately scored, in order to facilitate identification of those that are closest to the native structure [5]. The sampling and scoring approaches can also be applied in an iterative cycle, in order to find a pathway towards a more consistent refinement. However, both the sampling and scoring of improved models remains elusive, and the consistent refinement of predicted 3D models has not yet been witnessed in the CASP experiments. The refinement category itself has seen more limited success in the CASP experiments, compared with the tertiary structure prediction and quality assessment categories [5,53,54]. However, it must be emphasised that the refinement of the typical predicted 3D models produced by standard prediction servers is often much more successful than the refinement of the models selected by the CASP assessors for the refinement category, as the CASP “refinement targets” may have already been refined during other modelling pipelines [54,58].

In the following sections, we will outline the alternative methods used for both sampling and scoring. We will describe, compare, and contrast the different strategies and discuss the merits and pitfalls of each approach.

## 2. Sampling Strategies

Two broad approaches are used in the sampling stage: the fully-automated server-based programs and the non-server-based, highly central processing unit (CPU)-intensive programs, such as Molecular Dynamics (MD) simulations (also known as manual/human refinement methods in CASP) [43,64,65]. The sampling approaches may include the various combinations of knowledge-based methods [32,41,47,52,64,66,67,68,69,70,71,72], Monte Carlo simulations [68,69,70,73,74,75,76,77], physics-based potentials [69,70,78,79,80,81,82,83], and MD simulations [32,43,48,79,80,81,82,83,84,85,86,87,88,89,90,91,92,93], in order to sample near-native conformations.

Automated and rapid server-based refinement methods are generally based on side-chain optimisation and energy minimisation. Server-based approaches are practical, as they are often based on utilising the knowledge of protein structures, particularly specific interactions between residues and atoms, and they require less computational effort [43,56,57,58]. The generation of 3D models with automated server-based strategies is often more conservative and risk-averse, compared to the more computationally-intensive manual approaches, which often utilise MD-based approaches, as seen in the recent CASP experiments. Furthermore, the more conservative servers performed well in both CASP8 and CASP9, and the structural deviations among the generated sampled models were not as great as those observed in sampled models from the more computationally-intensive manual approaches [56,57,58,89]). On the other hand, these early conservative servers were not as successful as the non-server MD-based methods in the cases where the starting models were of poor quality, and where there was more room for improvement [5,53,64,65,83].

Since CASP10, the non-server-based highly CPU intensive methods, which have mainly relied upon MD simulations using physics-based force fields, parallel computing on graphics processing units (GPUs) and/or CPUs, and smart constraints, have become more widely-used to generate sample 3D models that are closer to the native structures [5,53,64,65,94]. MD simulations also provide important information about dynamic aspects of the structure [29,32,48,69,80].

A leading MD-based refinement approach, using a physics-based potential, was developed by the Shaw group [90,91,95] and tested in CASP9. However, they used a simulation time of 100 µs for each target, which was subsequently found to be unnecessarily long. Furthermore, structural deviations were also observed due to force-field inaccuracies and the lack of guidance towards the native basin during MD simulations [48,90,91,95].

In CASP10, the Feig group also developed a physics-based sampling approach using MD simulations, and managed to refine large proteins with shorter simulation times [32]. The MD-based protocol from the Feig group made significant progress towards a more consistent refinement with the usage of an improved force field, the application of C-alpha restraints, and an ensemble averaging stage under explicit solvent conditions [32,64]. However, the approach used by Feig was still extremely CPU intensive, requiring 75,000 core hours (12 days on 256 cores) to refine a single 3D model, and so it was not broadly applicable for the sort of large-scale analysis typically required by servers or proteomic pipelines [32].

With the growing availability of GPU/CPU computing [55,96], most of the top-performing groups in CASP12 also used MD-based sampling strategies [48,53,87,96,97,98,99,100,101]. Nevertheless, the sampling of alternative refinement models through MD simulations still brings about a high computational cost, particularly for large protein targets. Additionally, there remains a need for improved force fields to consistently increase the accuracy beyond that of the starting model, particularly where the starting model is already of high accuracy [5].

Force field accuracy is an important component of molecular simulations, as the chosen force field determines how the potential atomic interactions are modelled in molecular systems. The optimal parameters of force fields used in the simulations are determined from datasets of experimental structures [5,102]. Recently, popular force fields, such as the Chemistry at Harvard Macromolecular Mechanics (CHARMM) c22/CMAP [103] and c36 [97] versions and the AMBER ff14SB [99] and AMBER12SB [104,105] force fields, have been used in different sampling approaches, which included Monte Carlo and Molecular Dynamics simulations in the refinement pipeline [56,77,94,106]. However, all force fields are imperfect and cannot yet be relied upon to consistently generate models that are closer to experimental structures. There is plenty of room for improvement in force field development. Perhaps the main challenge is the further development of the parameter optimization strategies for the potential energy functions [32,48,69,78].

Due to the use of imperfect force fields, molecular dynamics simulations also suffer from lack of guidance for producing sample models that trend towards the native structure [69,78]. The usage of smart restraints has been a key factor in ensuring that the refinement models do not deviate away from the native structure [32,48]. However, there is a balance to be made, as the application of restraints may limit the extent of the refinement sampling; very strong restraints may just allow sampling of conformations that are close the starting model, instead of allowing a trend towards the native state [48]. Research has shown that the application of restraints is crucial, particularly where the initial model is highly accurate. It has also been observed that unrestrained MD simulations quickly drive the initial models away from the native structure [48,53,78,80,90,107]. Furthermore, the strength of the applied restraints has been found to be a significant parameter, in terms of increasing the quality of the sampled models, but it is interesting to note that weaker, rather than stronger, C-alpha restraints have often performed better [32,48,53,108].

**Figure 2:**
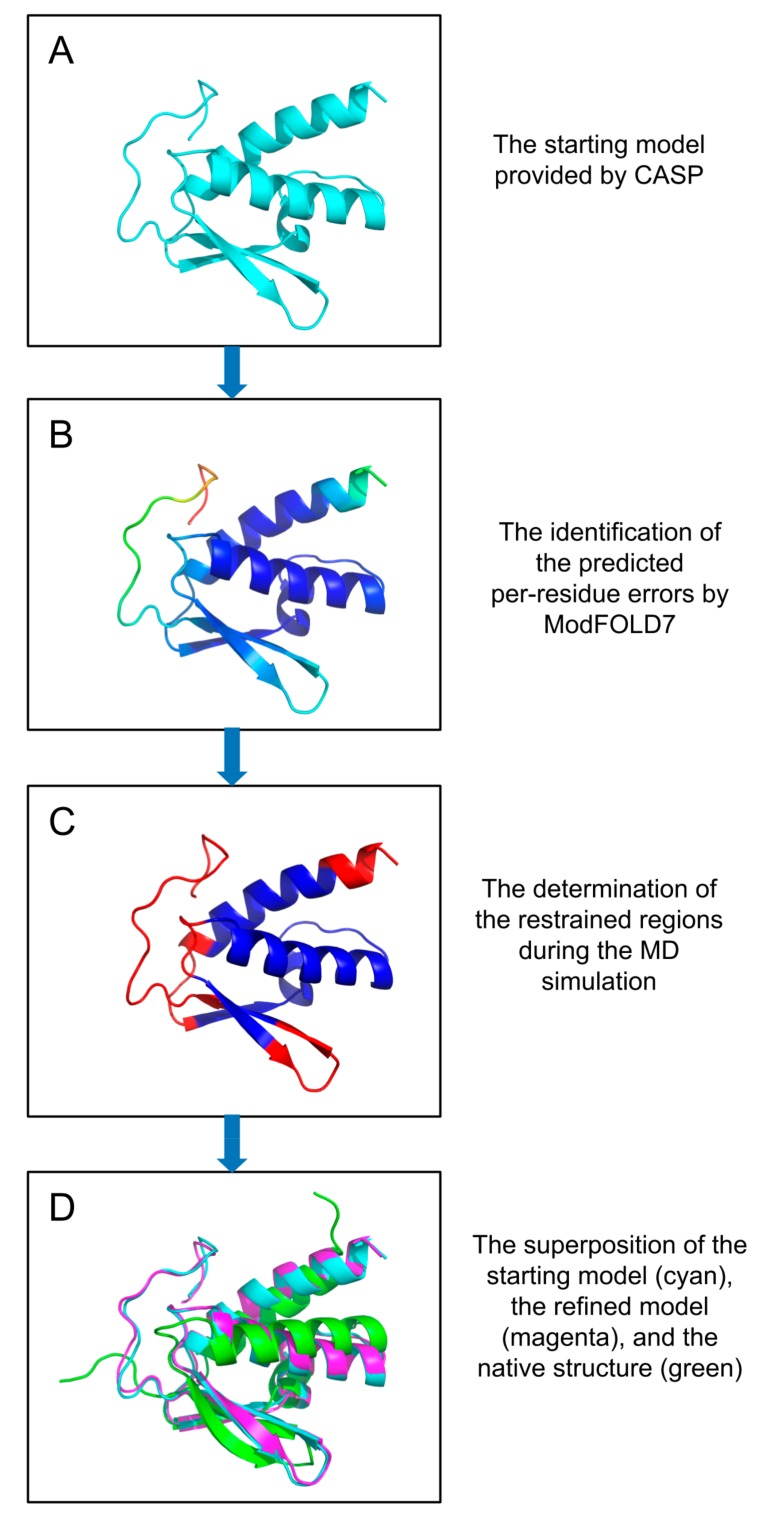
Example of refinement of a CASP13 model by the McGuffin group. The predicted per-residue error is produced by ModFOLD7 and, then, a new restraint strategy, based on the predicted per-residue error, is applied during the sampling stage: (**A**) CASP13 prediction target T0958; (**B**) top selected server model (BAKER-ROSETTASERVER_TS2), displayed using the B-factor scheme; (**C**) the top selected server model is coloured using an occupancy column, where blue regions indicate restrained residues and red regions indicate unrestrained residues during the MD simulation; (**D**) superposition of the top selected server model (cyan), refined model (magenta), and native structure (green). T0958: BAKER-ROSETTASERVER_TS2 versus T0958_ReFOLD_8, a GDT_HA improvement from 0.419 to 0.4464.

In most cases, the restraints have generally been applied on all C-alphas, but different kinds of restraints, based on prior knowledge [5,81,109], specific regions [5,81,109,110], and local quality assessment [5,88,111], have also been applied by groups participating in CASP experiments. The application of partial restraints can also give the sampling approaches more “wiggle room” to improve the quality beyond that of the initial models. The determination of which specific parts of a model are in need of more refinement, based, for example, on local quality estimates, may provide more reliable guidance for MD simulations [88,111,112]. Based on this principle, our group (the McGuffin group) has developed a new local quality assessment guided restraint strategy, which we used in CASP13. The strategy depends on the predicted per-residue accuracy scores produced by ModFOLD7. The regions of the starting models that are predicted to be close to the native structure are used as restraints for the MD simulations (Figure 2). Flat-bottom potential widths of 2–4 Å were also applied by the Feig group in CASP13, as a new restraint strategy which performed better than weak harmonic positional restraints [94,113]. The new restraint strategies that were applied in CASP13 showed a promising step towards a more consistent refinement.

The predicted residue–residue contacts have also made significant improvements to protein structure prediction strategies, particularly during the CASP13 experiment [114,115]. This valuable information has helped to increase the accuracy of the predicted 3D models. Furthermore, accurate information regarding predicted pairwise distances might also provide very valuable guidance for a more consistent refinement.

### Sampling Protocols

The refinement sampling strategies, described above, have been developed by expert groups participating in the CASP experiment and most of the more intensive methods are not straight-forward to deploy for general biologists. However, many of the groups have also developed web servers and/or stand-alone tools, many of which are freely available and easily accessible for life scientists who wish to apply 3D models to understand different molecular systems (see Table 1). Feig [5] has also provided a thorough review of the MD-based sampling strategies. 

PREFMD is a refinement web server based on the successful MD-based strategy tested in CASP11 by the Feig group [85]. The locPREFMD web server, which was also developed by Feig group, aims at improving the local quality of predicted 3D structures, rather than the overall quality, with the molecular dynamics simulations using modified force fields, according to the MolProbity score [86]. 

The Rosetta hybridization refinement protocol, developed by the Baker group, was tested in CASP11 and CASP12 and performed well [77]. The refinement approach used is dependent on the accuracy of the starting models (high or low resolution) [77]. The high-resolution protocol consists of the refinement of the local regions, including the errors. If the starting models are predicted to be far away from the native state, then the whole structure is refined using the low-resolution protocol [77].

The Seok group has developed their GalaxyRefine method as a web server and its protocol is based on re-packing side chains and then repeated structural relaxation by short molecular-dynamics simulations [54,88]. The approach was tested in CASP8, CASP9, and CASP10, and it managed to improve the local and global quality of the starting models [54]. GalaxyRefineComplex was also developed in order to refine protein-protein interactions, based on the GalaxyRefine protocol [54,116].

The KoBaMIN refinement web server also employs an efficient protocol, based on the principle of energy minimisation using a knowledge-based force field [66]. The approach performed well in CASP8, CASP9, and CASP10, but mostly made conservative changes to the starting models [57,58,66,72].

The Floudas group developed the Princenton_TIGRESS server, which employs a combination of various restraint strategies: CYANA in the sampling stage [117], Rosetta Fast Relax relaxation [75], CHARMM in the short MD stage [84,102], and a machine learning approach in the selection step using ddFIRE [118], Banch [119], and Rosetta [75,120] energy functions, under implicit-solvent conditions [89]. The web server was subsequently upgraded (Princenton_TIGRESS2.0) with Support Vector Machine (SVM)-driven classification and enhanced MD stages [56]. The Floudas group methods were among the top five refinement programs in CASP10 and CASP11 [53,65,89].

The refinement of protein structure models is also possible using the ModRefiner algorithm, which is based on two main steps [67]: The first step is the refinement of the backbone topology, starting from C-alpha traces. This step is, then, followed by side-chain addition, using a physics- and knowledge-based force field [67].

3Drefine is based on the optimisation of hydrogen bonds network with MESHI [121] and atomic-level energy minimisation using composite physics and a knowledge-based force field [41,122]. The approach was tested in the CASP8 and CASP9 refinement categories, where it ranked among the top groups. The method uses a relatively conservative approach for sampling models, making very minor alterations to the backbone. i3Drefine is an iterative version of the 3Drefine refinement protocol, and is also presented as a web server [41,52,122].

The ReFOLD server, developed by our group, uses a unique hybrid approach consisting of three stages to refine 3D models and fix the errors identified by ModFOLD6 [112]. The first stage is based on the optimisation of hydrogen bonds and contacts using i3Drefine [43,52]. The second stage uses a scalable molecular dynamics simulation of the predicted 3D models with Nanoscale Molecular Dynamics (NAMD) [123]. In the final stage, ModFOLD6 is also used to evaluate and score the 3D models generated by the i3Drefine and NAMD protocols by giving predicted local and global errors [43,52,112,123]. The ReFOLD server was first tested in CASP12 and showed promising performance as a computationally efficient approach. The amino acid sequence and a 3D model (in Protein Data Bank (PDB) format) of the target are the only required inputs to refine protein structures and the method has recently been integrated with the IntFOLD server [124].

The original ReFOLD protocol was relatively novel, in that it used the model quality estimation method ModFOLD6 for scoring the sampled models, instead of energy functions. The protocol has now been further developed (ReFOLD2) with the guidance of the local quality assessment score produced by ModFOLD7 (see Figure 2). The developed approach was also tested in CASP13 and ranked among the top 10 refinement methods, according to its cumulative Global Distance Test Total Score (GDT-TS) score [43,112]. The following section discusses the alternative strategies which have been deployed by groups for scoring sampled models.

## 3. Scoring Strategies

The MD-based and knowledge-based sampling approaches, described above, generate numerous 3D models in different alternative conformations [83,96]. Therefore, in the next stage of the refinement process, it is necessary to be able to reliably score the alternative 3D models, in order to select those that are closer to the native structure than the starting model. However, the generated alternative models are often very similar to one another, and this represents a challenge for developers of energy functions and/or quality assessment tools [5,48,54,71,83,88,108,110,125,126,127,128,129,130,131,132,133].

In Anfinsen’s hypothesis, it is stated that the native state has usually been found at the lowest Gibbs free energy, and native-like conformations are represented at a lower energy [126,134,135]. In further analysis, the most native-like state was found generally to be at the lowest energy score comparing to other states, but not always [94]. 

To score the 3D models sampled by the MD-based approaches utilising CHARMM c36 [97] and AMBER ff14SB force fields [99], several different energy functions have been tested to select native-like structures. Energy functions derived from the statistical analysis of known structures typically have been utilised to recognise native and native-like structures in the refinement; for example, the DFIRE [118], DDFIRE [118], RW+ [134], and Rosetta energy functions [5,48,108,126,136,137,138]. The energy scoring methods vary, depending on the choice of the reference state used to statistically analyse the atomic interactions based on known structures [48,83,108,126,139,140,141,142,143,144,145,146,147,148]. The lowest score produced by the scoring methods correlates with the lowest Root Mean Square Deviation (RMSD) score, but a consistent selection and a clear correlation is still required [55,94,106,134,149,150].

The distance-scaled, finite-ideal gas reference (DFIRE) [118,151,152] is one of the knowledge-based statistical potentials used to score native-like structures, using a distance-dependent and pairwise statistical energy function to find the 3D models closer to the native state. The lowest DFIRE score is often used to select the most native-like structures from among alternatives 3D models generated by the MD-based protocols, but it was not better than the final MD structure [48,118,151,152].

Random Walk reference state (RWplus) [134] scoring has also been used to score native-like structures. The RWplus score is based on a knowledge-based potential, including distance- and orientation-dependent potentials trained using databases of known structures [55,134]. The performance of the RWplus score was found to be better than the DFIRE score, in terms of the selection of native-like structures in refinement pipelines [55]. 

Rosetta energy functions [126] often identify the native-like states at a lower energy than the non-native structures [74,75,120,137,138,153,154,155]. Therefore, Rosetta energy function searches are often performed to discover the lowest energy conformation among the 3D models generated by the sampling approaches. [156]. The Rosetta energy function was also used to score the 3D models by the Baker and Feig groups in CASP13 [113,126]. However, energy-based approaches for selecting native-like conformations have not shown considerable improvement in recent years [126,157].

More recently, MQAPs, such as ProQ [158], ProQ2 [159], SELECTpro [160], and ModFOLD6 [112], have also been used to identify the most native-like structures, following the sampling stages in the refinement pipeline [43,72,106]. The MQAP approaches have traditionally been used for selection of the best models from among those submitted by tertiary structure prediction servers in the CASP experiments. In this role, they have performed well, in terms of selection of the most native-like predicted 3D models; furthermore, they are improving in their consistency [42,161,162,163]. However, such tools have not reached consistent selection for 3D models generated by refinement pipelines, where there is often much less variation. The consistent and accurate identification of the most native-like refinement models is a much harder task for MQAP methods, given the very small differences between models and, traditionally, MQAPs have not been developed for this specific role. 

## 4. CASP: The Critical Assessment of Techniques for Protein Structure Prediction

Evaluation of predicted protein structures from a wide range of prediction approaches requires objective blind tests, which are based on unreleased experimental structures [164]. The Critical Assessment of Techniques for Protein Structure Prediction (CASP) experiment has fulfilled the need for such objective testing since 1994. For more than two decades, John Moult and his colleagues have organised blind prediction experiments, every other year, in order to evaluate different approaches for various aspects of predicting structures from amino acid sequences [17,25,165]. The assessment experiment is always carried out by independent assessors and no prediction groups have access to the experimentally-determined structures for targets, prior to their release into the PDB [23,26,165,166]. 

### 4.1. The Refinement Category in CASP Experiments

The refinement category was introduced as an additional prediction category in CASP8, in order to encourage further improvements to the accuracy of predicted 3D models. The CASP assessors have typically provided the best predicted 3D models as refinement targets, in order to evaluate whether or not they can be successfully further improved [57]. Only the refinement of the provided starting model is requested and teams are discouraged from providing alternative models built from additional templates. The category aims to further increase in the accuracy of the best-predicted 3D models and the refinement methods have been able to add value to the prediction process [53,57,58,64,65].

It has been challenging for developers of refinement methods to improve the 3D models provided in the refinement category of CASP. This is primarily because the best-predicted models that are chosen as refinement targets may have already been once-refined in their source pipelines. Therefore, any further improvements to the quality of the provided models are, perhaps, less obvious and so it becomes an exercise of diminishing returns [57,58]. Moreover, some parts of the provided starting 3D models may have been based on known structures, particularly TBM predictions, and so the starting models might already be highly accurate and fairly close enough to the native structures [57,58]. Therefore, any “refining” of the starting models may be more likely to lead to deterioration in model quality, instead. With regard to the above, it is far harder to improve the quality of the predicted 3D models generated by TBM, compared to FM targets, as TBM models are often already highly accurate. In other words, the refinement of provided starting models that are already far away from the native structure are much easier to improve, and they more likely to improve in any refinement process, as there is more room for improvement to be made [5,57,58,64,83]. 

The selection of the CASP targets is also an important factor affecting the success of the refinement approaches. Small domains and domains that are free of crystal and oligomeric contacts have been preferred in previous CASP experiments [58]. Nevertheless, it is problematic to identify the target difficulties and compare performances across CASP refinement datasets [64]. For example, relatively bigger and oligomeric structures were selected as some of the refinement targets in CASP13, and such targets were far harder to refine than single small domains provided in previous CASPs.

The assessment criteria of CASP in the refinement category are mainly based on the comparison of the predicted 3D models with the native structure, utilising a wide range of measurements [64]. The alpha-carbon geometry and the backbone distance of the predicted models with the native structure are also the major component of the measurements based on superposition, particularly in the Template Modeling (TM)-Score [167]. Short-range contacts, including side-chain interactions, van der Waals clashes, and different elements in the structure are also taken into account by using the Ramachandran map along with the backbone units [57,58]. CASP assessors measure the global quality of predicted and refined models using the Global Distance Test (GDT) [168,169] (GDT_TS and GDT_HA) scores, and the Root Mean Square Deviation (RMSD) score, based on C-alpha atom superposition [57,58,167,168]. To measure the local quality of the models, the MolProbity [170] and SphereGrinder [171] (SphGr) scores have been used. The Local Distance Difference Test (LDDT) [172] score has also been used as a local and superposition-free measurement since CASP11 [65]. The global and local scores are combined into a weighted Z-score, in order to rank the models. The Z-score has been upgraded, using a machine learning algorithm, a Contact Area Difference Score [173] (CAD), and a Quality Control Score [174] (QCS), to compare performance in CASP12 [53].

It should be noted that the protein structures are flexible and can be observed in different conformations. The flexibility of the protein structures is a vital concept to consider, in terms of their functions; however, flexible regions are often not considered in CASP evaluations [58,175,176,177]. Although the experimental structures determined by NMR, X-ray crystallography, and cryo-electron microscopy represent an average conformation, average conformations are not perfect enough to justify their use in refinement approaches [58,178,179,180,181]. Therefore, non-native dependent measurements, such as the MolProbity score, could be considered more in the Z-score formula. Furthermore, the major CASP measurements, such as GDT-TS, GDT-HA, and RMS_CA, rely on backbone superposition, but the rate of the side-chain and local interactions could also be given more emphasis in the formula, depending on the interactions in the targets [53,57,58,64,65]. 

The refinement prediction groups in CASP are asked to submit up to five predicted or refined models, from the best to the worst under time constraints, and the first submitted model is assumed as the best model chosen by each group [53,57,58,64,65]. Submitting five predictions also enables groups to test different sampling approaches. In the CASP9 experiment, it was noticed that the prediction groups often had difficulties in ranking their structures accurately, and there were just a couple of groups who were able to rank their models better than a random selection [58]. Therefore, CASP assessors developed a new assessment method, called “cherry-picking”, as a second set of analysis [58]. The cherry-picked analysis considered the overall score as the best model, due to the lack of an accurate order of submitted models. However, accurate rank order of predictions is an important part of any 3D model selection process [58]. For example, MD-based approaches generate hundreds of models, so it is necessary to be able to accurately order the models for practical purposes. This issue highlights the importance of the scoring stage, but, presently, the CASP assessors do not evaluate the sampling and scoring methods independently in the refinement category. The need for identifying the best model was also emphasised in the following CASP experiments [53,58,64,65].

The sampling and scoring stages are different processes, and the best sampling or scoring groups have not been clearly distinguished in recent CASP experiments [57,58]. If prediction groups were to be able to submit more models, besides the top five models, then refinement methods could perhaps be assessed in terms of the sampling and scoring aspects. Such a separation of evaluation may help to boost the improvement of refinement methods. The relationship between sampling and scoring is complicated, and a strong correlation has not been found between observed scores and the available scoring methods [58]. Nevertheless, submitting additional models would bring an additional workload for CASP predictors and assessors; thus, a more pragmatic strategy may need to be devised. 

### 4.2. Progress with Refinement Strategies

It is noteworthy that, in the last 12 years, significant progress has been witnessed in the refinement category, since it was introduced in CASP8 [57]. However, initially, the top groups in CASP8 did not make any measurable improvement in performance in CASP9 [57,58]. It was also reported that the refinement approaches tested in CASP9 were found to be conservative, in terms of improving the starting models, and were not successful at correctly ranking the order of the submitted five models [57,58]. In CASP9, some hints from the assessors about accurate and problematic regions and the GDT-HA and GDT-TS scores of the starting models were also shared with prediction groups during the CASP experiment [58], although it is not known how many groups made good use of this information.

Although the cherry-picking approach was taken into consideration while analysing the performance of the refinement groups participating in CASP9, significant progress was not observed [58]. The overall score of the refined models was much lower than the starting models in CASP9 [58]. It was also observed that the conservative strategies were less likely to worsen the starting models than the more adventurous MD-based strategies. On the other hand, some of the MD-based approaches tested in CASP9 showed promising performance, in terms of sampling [58].

In CASP10, the leading groups managed to increase the accuracy of the backbone and side-chain interactions in most of the refinement targets [64]. However, the overall performance of most of the groups indicated that they were not able to consistently improve upon the starting models. The groups using MD-based approaches with access to advanced supercomputer facilities have opened a new epoch in the refinement of protein structures since CASP10, and they have generally performed much better than the knowledge-based approaches [64]. Significant energy changes were also observed among models generated by the more adventurous MD groups in CASP10, and energy scoring appeared to be more worthwhile information to be utilised by the scoring methods [64]. The top five groups also managed to improve their methods in CASP11 with the same pace gained in CASP10 [64,65]. Furthermore, the majority of the groups had improved more than half of the refinement targets in CASP11 [64,65].

While a modest improvement was seen in CASP8 and CASP9, compared to CASP10 [57,58,64], the progress in the MD-based approaches has led to successive gains in accuracy since CASP10 [65]. The growing trend in the consistency of the refinement of 3D models has been consolidated in CASP11 and CASP12 [53,65]. Although the targets were difficult, the refinement approaches tested in CASP12 have shown a considerable improvement over CASP11 [53]. The diversity of the refinement approaches in CASP12 is also promising for the future of the refinement, [53,166]. The numbers of targets and groups have increased dramatically since CASP8, from 12 to 42 targets and from 24 to 39 prediction groups in CASP12 [53,58,64,65]. In CASP13, many new hybrid refinement protocols emerged, using new restraint strategies and scoring functions, including energy functions and MQAPs [113]. These new methods performed well, in terms of increasing the accuracy of initial models, although the refinement targets were larger and more difficult, compared to previous CASPs.

One of the headline-grabbing groups from CASP13 was DeepMind, with their AlphaFold method for template-free modelling [182] however, the group did not participate in the refinement category. The success of the group in the free modelling category was partly due their accurate prediction of inter-residue distances. These more precise predictions could be used to enhance contact-based restraints in future refinement strategies.

## 5. Conclusions

The accuracy of 3D predicted models is a key factor for furthering *in silico* studies, particularly where experimental knowledge is scarce. Near-experimental accuracy is often required to properly understand the functional role of a protein, and the accuracy degree may vary, depending on the type of the computational application. Building 3D models with TBM and FM methods may not always be adequate to meet the required accuracy level for some biological applications, due to the unavailability of a suitable template and modelling errors, including irregular bonds and angles. Therefore, the refinement of predicted 3D structure is crucial for increasing the accuracy of initial structures and correction of local errors. Unfortunately, it is still challenging to deliver consistent refinement of 3D protein models, especially at high resolutions, as there is less room for improving the already highly-accurate predicted structures. The refinement of predicted 3D models consists of two independent stages—the sampling and scoring of refined models—and both should be the focus of future assessments, in order for us to gauge where progress is being made.

In the sampling stage, many different strategies, from rapid automated servers to highly computationally-intensive MD methods, have been suggested for improving initial structures towards the native basin. The MD-based sampling strategies have the potential to reach near-experimental accuracies with improvements in computing power and scoring methods. Unfortunately, the most successful approaches still require supercomputer-scale resources, which makes them less practical and may put them out of reach of general biologists.

Although the current force fields perform well, in terms of directing the initial structures towards the native structure, structural deviations are often encountered in MD simulations, due to imperfections. A wide range of restraint strategies, based on the knowledge of the native structures, have been applied to avoid structural deviations. The partial restraints, particularly based on known structures, may provide more reliable guidance for protein model refinement towards the native basin, compared to restraining the whole structure, as the application of restraints on poorly-predicted regions may limit the scope for refinement. For instance, the local quality assessment scores produced by MQAPs can provide an alternative approach for determining poorly-predicted regions, which could lead to more focused refinement, instead of refining or restraining the whole structures [43,77,88,111,112,156,183,184].

There are a few groups in CASP who start from sequences to build 3D models, assess the 3D models, and finally refine the best predictions. Our group (the McGuffin group) is one of the leading groups, in terms of producing local quality assessment scores, and our local quality assessment score is used to guide our short and fast MD-based refinement approach, which we tested in CASP13. The approach (ReFOLD2) is perhaps the first attempt at using local quality assessment scores to guide the MD simulation and assess the sampled 3D models. The aim of this approach is to more consistently refine the predicted 3D models with far less computational effort, by using the guidance of the predicted per-residue errors.

The accuracy of the scoring functions, including energy functions and MQAPs, is crucial for successful prediction and refinement. The 3D models generated by the sampling approaches are structurally very similar and, so, consistently distinguishing the most native-like states from non-native conformations, using either energy functions or MQAPs, still remains an unsolved problem.

The prediction of protein structures is a step towards computational functional analyses, but interactions with ligands, ions, and proteins are also important for determining protein functions. Therefore, ideally, the refinement of 3D models should also include oligomeric states and protein–ligand complexes. In the real world, proteins are always interacting with various ligands, such as ions, inhibitors, and peptides. Therefore, the refinement of protein models might still be somewhat artificial, if they do not also consider more complete molecular systems.

## Figures and Tables

**Table 1:** Publicly-available refinement web servers, based on methods tested in the CASP experiments.

Name	URL
PREFMD [85]	http://feiglab.org/prefmd
locPREFMD [86]	http://feig.bch.msu.edu/web/services/locprefmd/
GalaxyRefine [54]	http://galaxy.seoklab.org/refine
KoBaMIN [66]	http://csb.stanford.edu/kobamin
Princeton_TIGRESS 2.0 [56]	http://atlas.engr.tamu.edu/refinement/
ModRefiner [67]	http://zhanglab.ccmb.med.umich.edu/ModRefiner
3DRefine [41,122]	http://sysbio.rnet.missouri.edu/3Drefine/
ReFOLD [43]	http://www.reading.ac.uk/bioinf/ReFOLD/
FG-MD [110]	http://zhanglab.ccmb.med.umich.edu/FG-MD/

## Abbreviations

NMR	Nuclear Magnetic Resonance
CPU	Central Processing Unit
GPU	graphics processing unit
Cryo-EM	cryo-electron microscopy
PDB	Protein Data Bank
CASP	Critical Assessment of techniques for Structure Prediction
CHARMM	Chemistry at Harvard Macromolecular Mechanics
SVM	Support Vector Machine
NAMD	Nanoscale Molecular Dynamics
LDDT	Local Distance Difference Test on All Atoms
TBM	Template-Based Modelling
FM	Free Modelling
MQAPs	Model Quality Assessment Programs
MD	Molecular Dynamics
DFIRE	Distance-Scaled, Finite-Ideal Gas Reference
DDFIRE	Dipolar Distance-Scaled, Ideal Gas Reference
RWplus	Random Walk reference state Plus
GDT-TS	Global Distance Test Total Score
GDT_HA	Global Distance Test High Accuracy
SphGr	SphereGrinder
RMSD	Root mean square deviation
TM-Score	Template Modeling Score
